# Investigation on the molecular mechanism of SPA interference with osteogenic differentiation of bone marrow mesenchymal stem cells

**DOI:** 10.1038/s41598-024-66502-2

**Published:** 2024-07-06

**Authors:** Hong-jie Wen, Shou-yan Zhu, Hua-gang Yang, Feng-yong Guo

**Affiliations:** 1 Department of Orthopaedic and Trauma, The Affiliated Hospital of Yunnan University, Kunming, China; 2 Department of Orthopaedic and Trauma, The Second People’s Hospital of Yunnan Province, Kunming, China

**Keywords:** Staphylococcus aureus protein A, Bone marrow derived mesenchymal stem cells, Biomarkers, Bioinformatics, Osteogenic differentiation, Diseases, Medical research

## Abstract

Binding of Staphylococcus aureus protein A (SPA) to osteoblasts induces apoptosis and inhibits bone formation. Bone marrow-derived mesenchymal stem cells (BMSCs) have the ability to differentiate into bone, fat and cartilage. Therefore, it was important to analyze the molecular mechanism of SPA on osteogenic differentiation. We introduced transcript sequence data to screen out differentially expressed genes (DEGs) related to SPA-interfered BMSC. Protein–protein interaction (PPI) network of DEGs was established to screen biomarkers associated with SPA-interfered BMSC. Receiver operating characteristic (ROC) curve was plotted to evaluate the ability of biomarkers to discriminate between two groups of samples. Finally, we performed GSEA and regulatory analysis based on biomarkers. We identified 321 DEGs. Subsequently, 6 biomarkers (*Cenpf, Kntc1, Nek2, Asf1b, Troap and Kif14*) were identified by hubba algorithm in PPI. ROC analysis showed that six biomarkers could clearly discriminate between normal differentiated and SPA-interfered BMSC. Moreover, we found that these biomarkers were mainly enriched in the pyrimidine metabolism pathway. We also constructed '71 circRNAs-14 miRNAs-5 mRNAs' and '10 lncRNAs-5 miRNAs-2 mRNAs' networks. *Kntc1* and *Asf1b* genes were associated with rno-miR-3571. *Nek2* and *Asf1b* genes were associated with rno-miR-497-5p. Finally, we found significantly lower expression of six biomarkers in the SPA-interfered group compared to the normal group by RT-qPCR. Overall, we obtained 6 biomarkers (*Cenpf, Kntc1, Nek2, Asf1b, Troap*, and *Kif14)* related to SPA-interfered BMSC, which provided a theoretical basis to explore the key factors of SPA affecting osteogenic differentiation.

## Introduction

Osteomyelitis is one of the most challenging and difficult diseases in orthopedics. Osteomyelitis can easily induce bone defects, bone nonunion, and other related diseases, leading to limb dysfunction, amputation, and even life-threatening, seriously threatening the physical and mental health of patients^1^. Therefore, it is important to thoroughly investigate the mechanism related to osteomyelitis and bone defects. Staphylococcus aureus is the most common pathogenic microorganism in osteomyelitis, which can cause increased inflammation and progressive bone destruction^2^. SPA, expressed in most S. aureus, is an important virulence factor in the cell wall of S. aureus that interacts with human immunoglobulins^3^. When SPA binds to osteoblasts, it has been reported to induce apoptosis and cell death, thereby inhibiting bone formation and mineralization^4^.

Bone marrow-derived mesenchymal stem cells (BMSCs) are considered a promising cellular resource and potential therapeutic tool for improving graft function and pathological recovery in a variety of diseases^5,6^. The development of osteomyelitis with bone defects is closely related to the reduced osteogenic differentiation capacity of BMSCs. It was found that during the development of osteomyelitis, SPA not only directly stimulates the apoptosis of osteoblasts in the focal area, which in turn causes bone destruction and bone loss in the focal area, but also downregulates the osteogenic differentiation ability of BMSCs and upregulates their lipogenic differentiation ability. However, the decrease in the differentiation ability of BMSCs into osteoblasts directly affects osteogenesis and bone union, ultimately leading to infected bone non-union or the development of bone defects^7^. Interactions of chemokines and chemokine receptors mediate the migration of mesenchymal stem cells to the injured site in the brain after hypoglossal nerve injury, suggesting that SPA plays a key role in the development of osteomyelitis^8^. Therefore, an in-depth study of the causes of reduced osteogenic differentiation of BMSCs under the effect of SPA is important for the treatment of osteomyelitis with bone defects.

In our previous study, we found that the occurrence of infectious bone defects is related to the osteogenic differentiation of BMSCs, and SPA inhibits the osteogenic differentiation of BMSCs, but the specific mechanism of action is unknown. In this study, by constructing an osteogenic differentiation cell model of BMSCs under the effect of SPA and performing bioinformatics analysis, we identified 6 biomarkers (*Cenpf, Kntc1, Nek2, Asf1b, Troap* and *Kif14*) related to SPA-interfered BMSC, which laid a theoretical foundation for exploring the key factors of SPA affecting osteogenic differentiation. And we tried to explore the specific mechanism of action by constructing non-coding RNA interaction network. This is a new theoretical basis and research direction for further understanding and treatment of osteomyelitis and bone defects.

## Materials and methods

### Cell culture and sequencing

All cells were obtained from the Cell Bank of the Chinese Academy of Sciences (Shanghai, China) and osteogenically differentiated for 14 d. Two groups were established, the experimental group treated with SPA (1 mg/mL)^9^ (Aladdin Corporation, Shanghai, China) and the control group not treated with SPA, each consisting of 3 samples. Total RNA was extracted from all samples using TRIzol reagent (Ambion, Austin, USA) according to the manufacturer's protocol. RNA quality and integrity were assessed using an RNA quality assay. Reverse transcription of RNA into cDNA was performed using a first-strand cDNA synthesis kit (Servicebio, Wuhan, China). The subsequent steps involved precise end filling of the cDNA fragments to create blunt ends, followed by the addition of an 'A' base at the 3' ends (A-tailing) to facilitate adapter ligation. Polymerase chain reaction (PCR) amplification was performed using junction primers to selectively enrich the cDNA fragments of interest. These carefully prepared cDNA libraries underwent rigorous quality control prior to sequencing on the Illumina platform, allowing for comprehensive transcriptomic analysis.

### Data processing

The whole transcript sequence data of 6 BMSCs form Rattus norvegicus was obtained, of which 3 normal differentiated and 3 SPA interfered samples. The raw sequencing reads were processed using Trimmomatic (version 0.36) to remove adapters and low quality sequences, resulting in clean data. The clean reads were aligned to the reference genome using hisat2 (version 2.1.0). Alignment results were quantified using samtools (version 0.1.19) and RNA was quantified based on genomic positions annotated in the reference. Principal component analysis (PCA) was performed on the processed data using the 'scatterplot3d' package in R to visualize clustering and variability between different sample groups.

### Analysis of differential genes

The ‘Deseq2’ R package^10^ was applied to mine differentially expressed genes (DEGs) between normal differentiated group and SPA interfered group. The *P* value < 0.05 & |log2fold change (FC)|> 2.5 was determined as the signifcance criteria. Volcano plot and heatmap were applied to show DEGs. Gene Ontology (GO) and Kyoto Encyclopedia of Genes and Genomes (KEGG) enrichment analysis of DEGs was performed using ‘clusterProfiler’ and KEGG pathway database was used for pathway enrichment (https://www.kegg.jp/kegg/)^11–14^. *p* < 0.05 was used as screening criteria.

### Protein–protein interaction (PPI) network

PPI network which depicted the interactions among DEGs was generated using search tool for the retrieval of interacting genes (STRING) website (https://string-db.org). Molecular Complex Detection (MCODE) algorithm of cytoscape plug-in was applied to screen core gene cluster^15^. Subsequently, we applied 4 distinct centrality measures provided by cytohubba plug-in: degree, edge filtered component (EPC), betweenness and closeness. These centrality measures are widely recognized for their utility in network analysis: 1. Degree centrality reflects the number of connections a node has and is commonly used to pinpoint nodes with high direct influence within the network. 2. EPC estimates the influence of a node based on the connection strength with its neighbors, highlighting genes capable of connecting various network regions. 3. Betweenness centrality considers the number of shortest paths passing through the node, thus identifying gene nodes that serve as critical bridges within the network structure. 4. Closeness centrality reveals nodes that can quickly interact with other nodes due to their shorter path lengths to all other nodes in the network. Eventually, the biomarkers for this study were obtained by overlapping 10 genes with the highest scores.

### Receiver operating characteristic (ROC) and gene set enrichment analysis (GSEA)

ROC curve was plotted to evaluate the ability of biomarkers to distinguish normal differentiated cells from SPA-interfered cells by ‘pROC’ package^16^. GSEA was conducted to explore the potential GO items and KEGG pathways associated with biomarkers through ‘clusterProfiler’ package^17^. *p*. adjust < 0.05 was used as screening criteria.

### The circRNA/lncRNA-miRNA-mRNA network construction

In order to explore the regulatory relationship, miRDB (https://mirdb.org/index.html) was utilized to forecast the miRNAs of biomarkers. circRNAs that interacted with miRNAs were predicted through the miranda (www.microrna.org / microrna / home.do) database. Further, circRNAs with consistent expression trends with biomarkers were applied for subsequent analysis. The same method was yielded to obtain the lncRNAs. Cytoscape software^18^ was applied to optimize the ‘circRNA-miRNA-mRNA’ and ‘lncRNA-miRNA-mRNA’ network.

### The analysis of the expression of biomarkers

In order to confirm the expression of biomarkers, we implemented Reverse Transcription quantitative-Polymerase Chain Reaction (RT-qPCR). The 5 normal SD-BMSC1 cells and 5 intervention cells were obtained with the consent from The Affiliated Hospital of Yunnan University, and this study was approved by the ethics committee of The Affiliated Hospital of Yunnan University. RNA extraction was performed by homogenizing the cells in TRIzol reagent, followed by phase separation with chloroform, RNA precipitation with isopropanol, washing with 75% ethanol, and resuspension in RNase-free water. The RNA quality and concentration were assessed using a NanoDrop spectrophotometer (Thermo Fisher Scientific, USA). Subsequently, RT-qPCR was conducted using the 2 × Universal Blue SYBR Green qPCR Master Mix (Servicebio, Wuhan, China), following the manual's guidelines. The PCR amplification was performed in a real-time PCR system (Bio-Rad, USA), with the thermal cycling conditions set as follows: initial denaturation at 95 °C for 10 min, followed by 40 cycles of 95 °C for 15 s and 60 °C for 60 s. The primer sequences for PCR were tabulated in Table 1. GAPDH was used as an internal reference gene, and the expression was calculated according to the 2^−ΔΔ^Ct method^19^.

**Table 1:** The primer sequences of biomarkers for RT-qPCR.

Primer	Sequences
Cenpf F	GTTTGAATCGCTCGTGCTGG
Cenpf R	TCCTTCCACTCTTCCAACGC
Kntc1 F	CTGAGAAGACACTGACGTGGA
Kntc1 R	CGAGACTCCGGTAAGTACGC
Nek2 F	GGCCTCAGCAGAAAGGGATT
Nek2 R	AGGAGTCTGCGTGTTTAGCC
Asf1b F	CCTGTCTGACGACCTTGAGTG
Asf1b R	GGTGCAGGTGATGAGAACCA
Troap F	GCTTGTCTCACCACCATCCA
Troap R	GGAATGAAACGCAGGGCATC
Kif14 F	CTCAGCGACCAATCGGGAAG
Kif14 R	CTCAGCCTACCGGCTCTCTG
GAPDH F	GACCCCTTCATTGACCTCAAC
GAPDH R	GCCATCACGCCACAGCTTTCC

### Statistical analysis

All P values of statistical results were based on two-sided statistical tests, and a *P* value < 0.05 was considered statistically significant.

## Results

### Identification of DEGs related to SPA interfered BMSC

**Figure 1:**
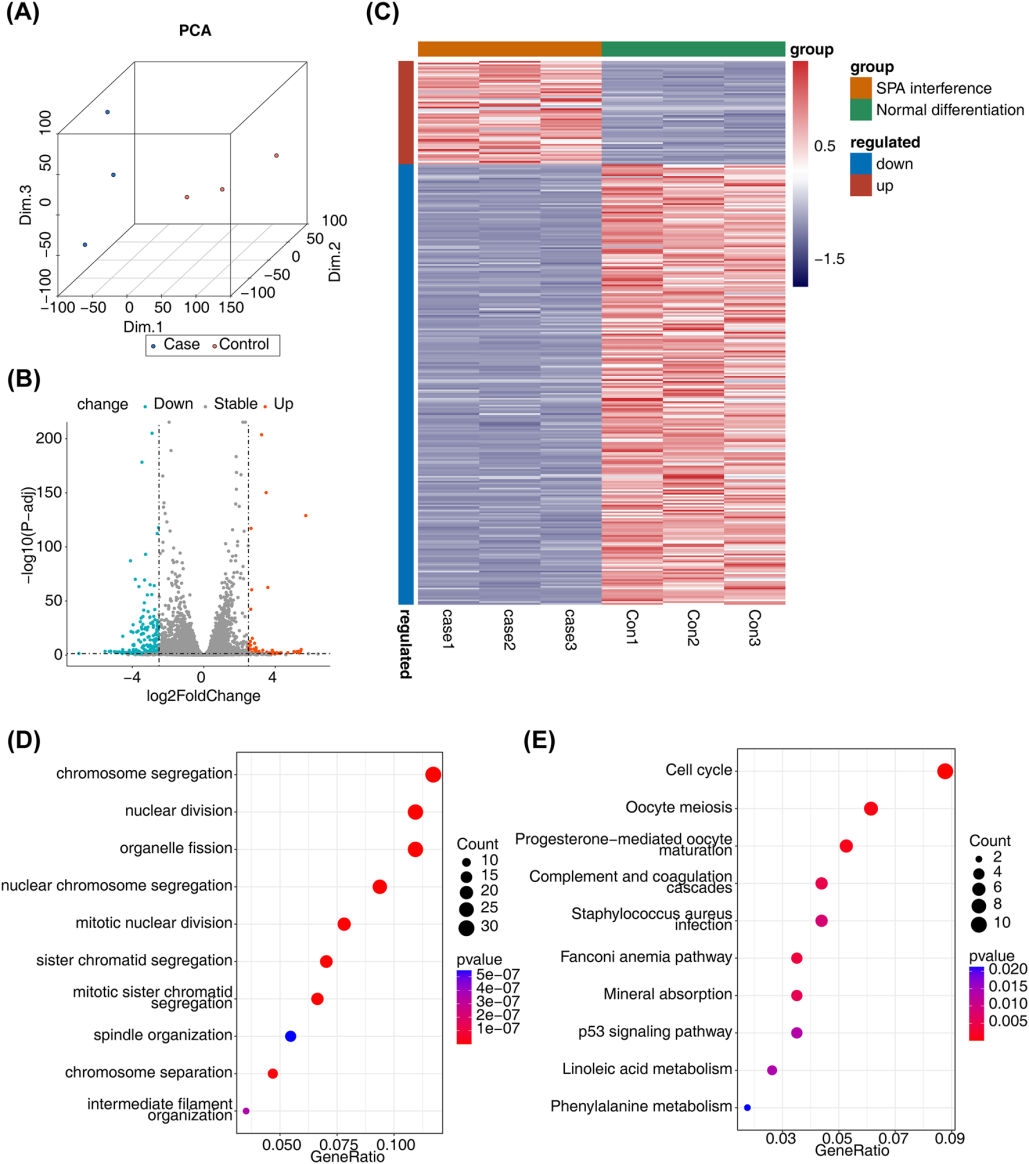
Identification of DEGs and functional enrichment analysis. (**A**) PCA analysis of normal differentiated group and SPA interfered group. (**B**, **C**) The volcano map (**B**) and heat map (**C**) of DEGs between normal differentiated group and SPA interfered group. (**D**, **E**) The GO terms (**D**) and KEGG pathways (**E**) enriched in DEGs. KEGG pathway database was used for pathway enrichment (https://www.kegg.jp/kegg/). DEGs, differentially expressed genes; PCA, principal component analysis; SPA, Staphylococcus aureus protein A; GO, gene ontology; KEGG, Kyoto Encyclopedia of Genes and Genomes.

Using the mouse reference genome alignment, the mapping rate of all samples was above 88.15%, indicating that the quality of sequencing was very good. PCA analysis suggested that there was obvious separation between the two sample groups (Fig. 1A). 321 DEGs were identified in SPA interfered vs normal differentiated group, including 260 down-regulated and 61 up-regulated genes (Fig. 1B, C). To further probe the function of the DEGs, functional enrichment analysis was conducted. GO results indicated that these DEGs were principally involved in ‘mitotic nuclear division’, ‘chromosome separation’ and ‘nuclear division’ (Fig. 1D). Additionally, the KEGG analysis demonstrated that these DEGs were mainly enriched in the ‘Complement and coagulation cascades’ and ‘p53 signaling pathway’ (Fig. 1E).

### Screening of biomarkers associated with SPA interfered BMSC

**Figure 2:**
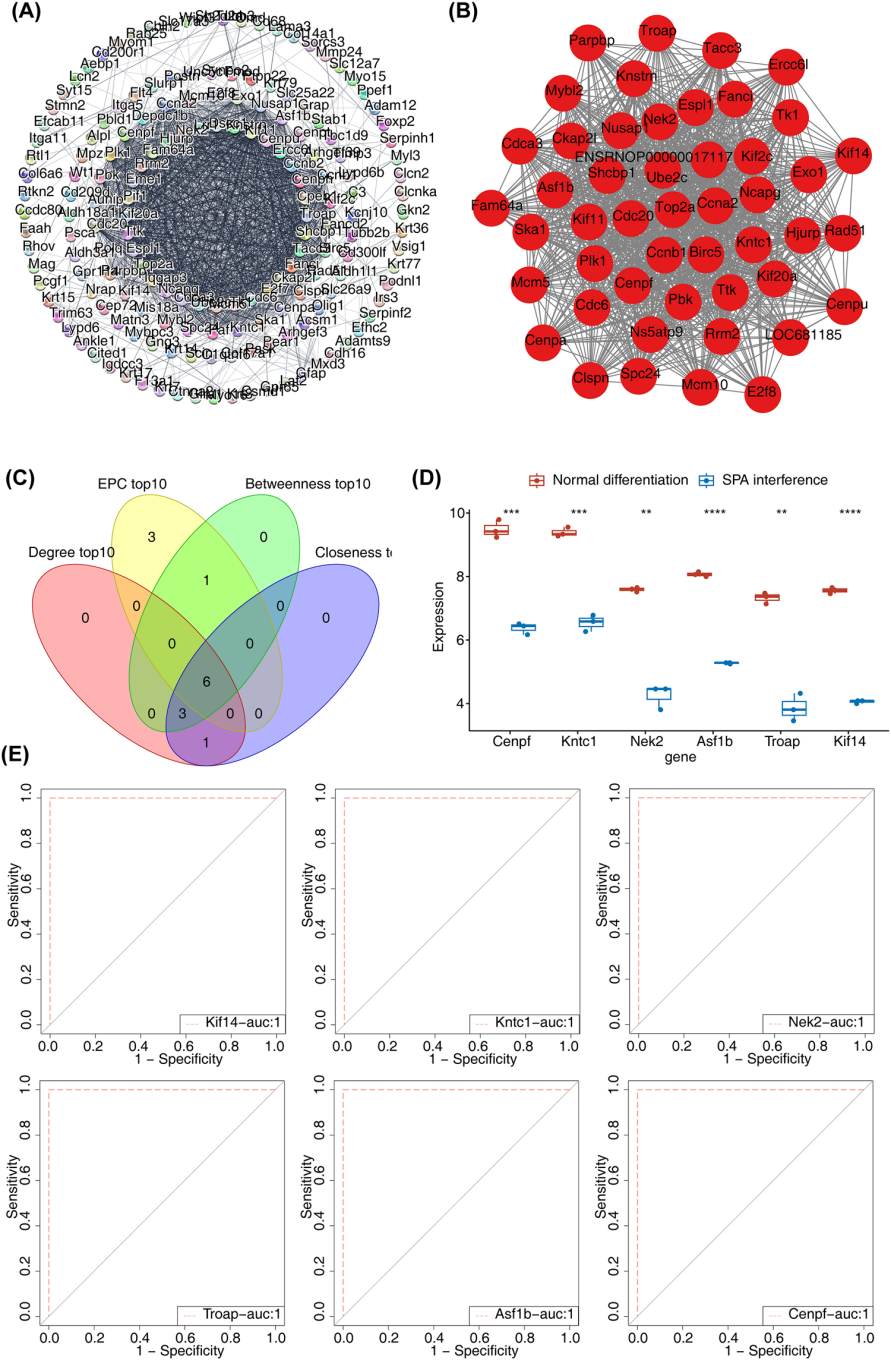
Identification of biomarkers for SPA interfered BMSC. (**A**) The PPI network of DEGs. (**B**) The interaction of the important gene cluster. (**C**) The Venn diagram of six biomarkers. (**D**) The expression of biomarkers in normal differentiated group and SPA interfered group. (**E**) The ROC curves of biomarkers. BMSC, bone marrow mesenchymal stem cells; PPI, protein–protein interaction; ROC, receiver operating characteristic; AUC, area under the curve. **p < 0.01; ***p < 0.001; ****p < 0.0001.

In order to explore the interaction regulation relationship, the PPI network of the DEGs was constructed, including 295 nodes and 1423 edges (Fig. 2A). We then screened an important gene cluster, which include 48 genes (Fig. 2B). After that, six biomarkers associated with SPA interference, including Cenpf, Kntc1, Nek2, Asf1b, Troap and Kif14, were obtained by overlapping 10 genes with the highest scores based on four algorithms in the 48 gene cluster (Fig. 2C). At the transcription level, we observed lower expression of Cenpf, Kntc1, Nek2, Asf1b, Troap, and Kif14 in SPA interfered group compared to the normal differentiated group (Fig. 2D). The area under the curve (AUC) values of biomarkers were all 1, indicating an excellent ability to distinguish normal differentiated cells from SPA-interfered cells (Fig. 2E).

### Functional enrichment analysis

**Figure 3:**
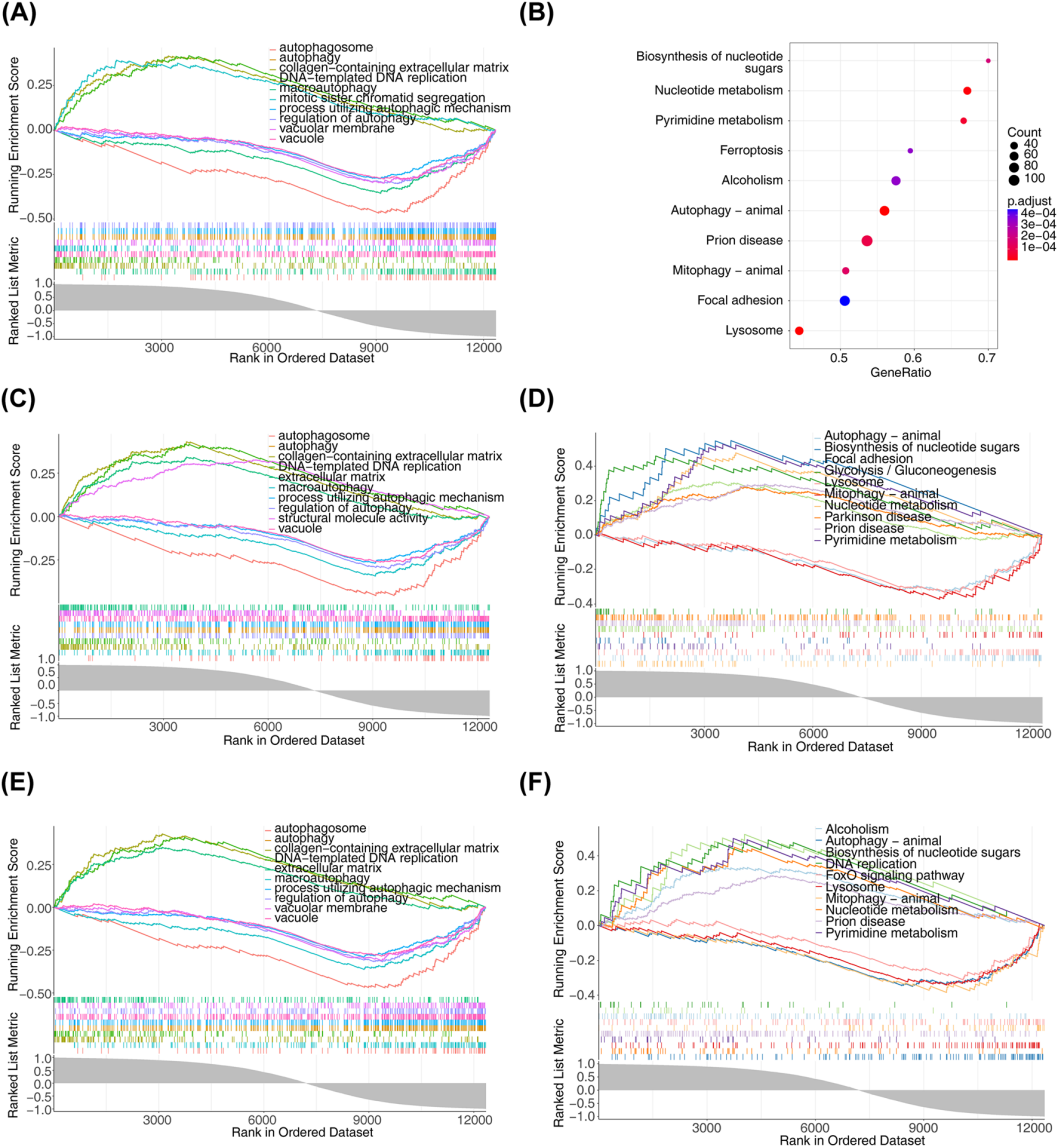
Functional enrichment analysis. GO terms and KEGG pathways enriched in Cenpf (**A**, **B**), Kntc1 (**C**, **D**), Nek2 (**E**, **F**). KEGG pathway database was used for pathway enrichment (https://www.kegg.jp/kegg/).

**Figure 4:**
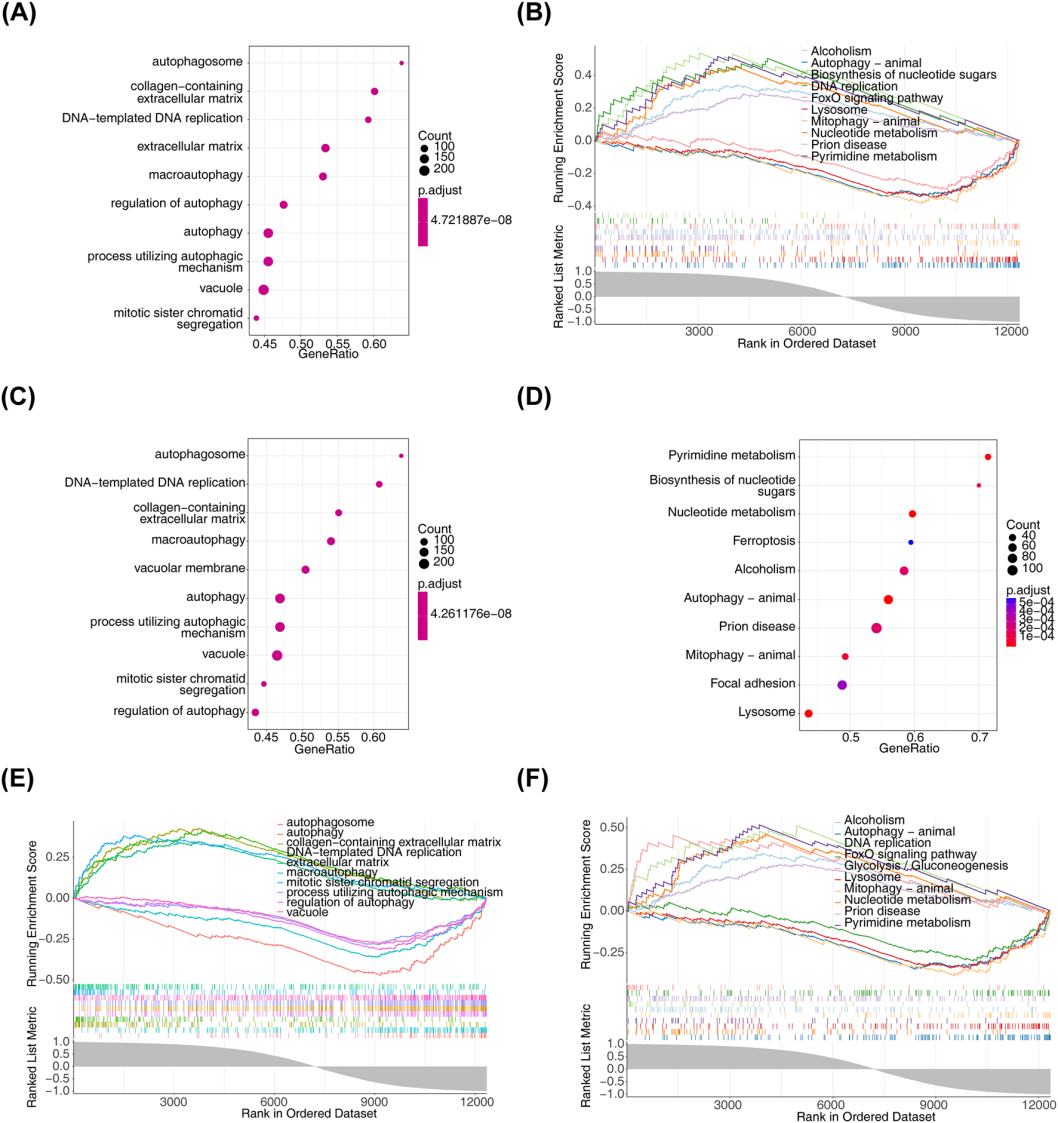
Functional enrichment analysis. GO terms and KEGG pathways enriched in Asf1b (**A**, **B**), Troap (**C**, **D**), and Kif14 (**E**, **F**). KEGG pathway database was used for pathway enrichment (https://www.kegg.jp/kegg/).

To further study the potential roles of Cenpf, *Kntc1, Nek2, Asf1b, Troap* and *Kif14* related to SPA interference in BMSC, we performed single-gene GSEA on biomarkers. The results showed that *Cenpf* was mainly enriched in the ‘regulation of autophagy’ and ‘Lysosome’ (Fig. 3A, B). *Kntc1* and *Nek2* were mainly related to the ‘autophagosome’ and ‘Pyrimidine metabolism’ (Fig. 3C–F). *Asf1b* and *Troap* was mainly enriched in the ‘process utilizing autophagic mechanism’ and ‘Biosynthesis of nucleotide sugars’ (Fig. 4A–D). *Kif14* was mainly enriched in the ‘macroautophagy’ and ‘Pyrimidine metabolism’ (Fig. 4E, F).

### Construction of the regulatory network

**Figure 5:**
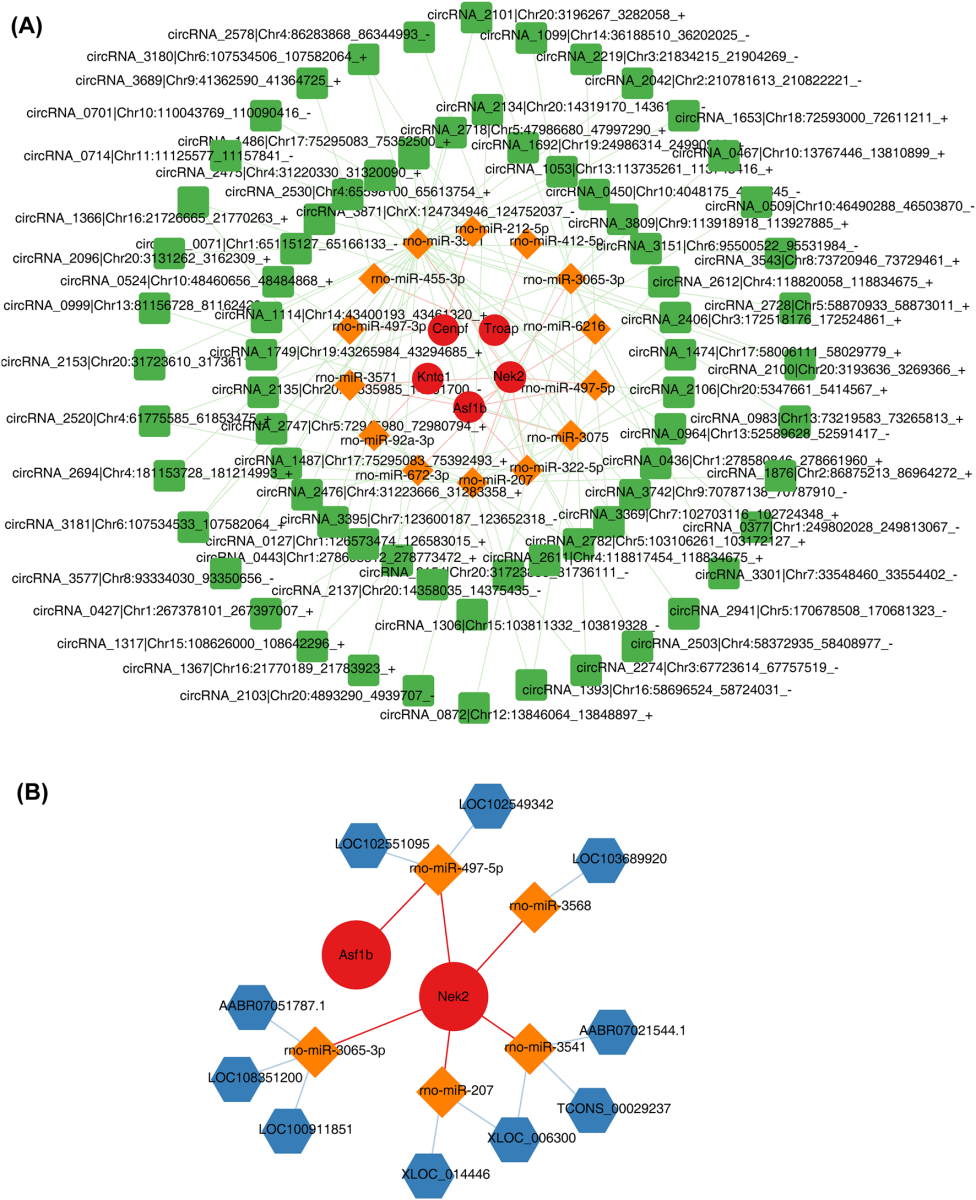
Exploration of regulatory mechanism for biomarkers. (**A**) The network of circRNA-miRNAs-mRNA.The green squares represent circRNAs, orange squares represent miRNAs, and red circles represent mRNA. (**B**) Construction of lncRNA-miRNA-mRNA network. Blue hexagons represent lncRNAs, orange squares represent miRNAs, and red circles represent mRNA. circRNA, circular RNA; miRNA, microRNA; lncRNA, long non-coding RNA.

To explore the regulatory mechanism of biomarkers associated with SPA interfered BMSC, 71 circRNAs-14 miRNAs-5 mRNAs network was constructed (Fig. 5A). The network had 90 nodes and 107 edges, in which *Kntc1*, and *Asf1b* genes were associated with rno-miR-3571. Additional, 10 lncRNAs-5 miRNAs-2 mRNAs network was constructed (Fig. 5B).The network had 34 nodes and 17 edges, in which *Nek2,* and *Asf1b* genes were associated with rno-miR-497-5p.

### Experimental verification of marker expression level

**Figure 6:**
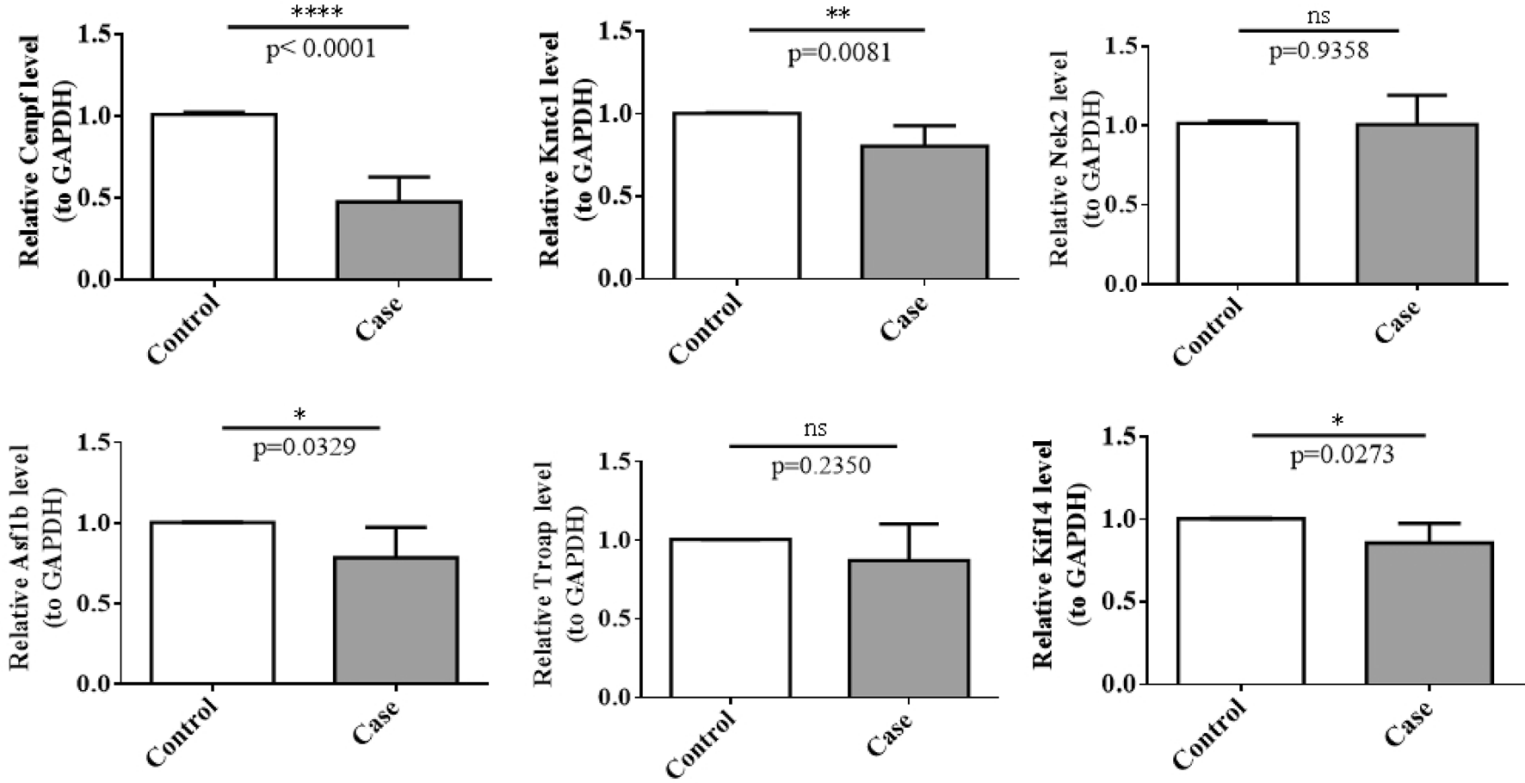
Validation of the expression of biomarkers by qRT-PCR. ns, not significant; *p < 0.05; **p < 0.01; ****p < 0.0001.

We verified the expression in clinical cell samples by RT-qPCR, which in agreement with the results of the public database data analysis. The expression of *Cenpf, Kntc1, Asf1b* and *Kif14* were notably reduced in clinical SPA interfered group versus normal group. However, no significant differences were observed between the two groups for *Nek2* and *Troap* (Fig. 6).

## Discussion

Our previous study and other related studies have shown that SPA can affect the osteogenic differentiation of BMSCs. In this study, bioinformatic analysis of transcriptome sequencing data revealed that the osteogenic differentiation of BMSCs under the effect of SPA caused the differential expression of molecular markers *Cenpf*, *Kntc1, Nek2, Asf1b, Troap* and *Kif14.* And these molecular biomarkers formed a network of interactions with nRNA. There were few studies on this aspect.

Kinetochore-associated 1 (*Kntc1*) encodes a protein that is one of many involved in mechanisms that ensure proper chromosome segregation during cell division. Functional enrichment analysis revealed that *Kntc1* was mainly associated with autophagosome and pyrimidine metabolism. Recent studies have suggested that mitogenic proteins may be potential biomarkers and may contribute to the development of human malignancies^20^. It has been frequently associated with tumors of the digestive and genitourinary systems^21^. It was shown that *Kntc1* was highly expressed in hepatocellular carcinoma (HCC) tissues and was associated with poor prognosis, suggesting a key role for *Kntc1* in HCC development^22^. Wnt signaling, MAPK signaling, c-Jun NH2-terminal kinase (JNK) signaling, PI3K/Akt signaling, Hedgehog signaling, and other signaling pathways are closely related to osteogenic differentiation^23–25^. Kntc1 has been reported to function in a variety of diseases by participating in the PI3K/Akt pathway^22^, and we speculated that it may also be involved in the regulation of osteogenic differentiation in BMSCs. *Kntc1* has been shown to play an important role in the PI3K/Akt signaling pathway^26^, which has been extensively studied and shown to play a key regulatory role in osteogenic differentiation. Therefore, *Kntc1* may regulate osteoblast differentiation and function through this pathway, a finding that is consistent with the performance of *Kntc1* in our results.

Centromere protein F (*Cenpf*) was a protein-coding gene. Functional enrichment analysis showed that *Cenpf* was mainly enriched in "regulation of autophagy" and "lysosome". Overexpression of *Cenpf* was associated with the tumorigenesis of many human malignant tumors^27–29^. Moreover, *Cenpf* was a cancer stem cell (CSC)-specific marker gene, and the latter played a key role in promoting bone destruction^30^. *Cenpf* has a close relationship with the MAPK^31,32^ and Wnt^33^ signaling pathways. Antisilencing function 1b (Asf1b) had effects on cell proliferation, leading to abnormal nuclear structure and unique transcriptional features^34^, and was frequently associated with various malignancies^35,36^. According to the functional enrichment analysis, *Asf1b* was mainly enriched in "process using autophagic mechanism" and "nucleotide sugar biosynthesis". In addition, several studies have shown that *Asf1b* plays an important role in the PI3K/Akt signaling pathway^37–39^. Never in mitosis gene A-related kinase 2 (*Nek2*) was highly associated with drug resistance, rapid recurrence and poor outcome in a variety of cancers^40^. Functional enrichment analysis showed that *Nek2* was mainly related to "autophagosome" and "pyrimidine metabolism". It has been shown that *Nek2* overexpression is associated with the development of bone damage^41^ and that it regulates osteoblast gene expression and affects osteoblast growth and activity^42^. In addition, *Nek2* induced osteoclast differentiation and bone destruction via heparanase in multiple myeloma^43^. *Nek2* has been reported to play an important regulatory role in MAPK^44,45^, Wnt/β-catenin^46–48^, PI3K/Akt^49^, and other signaling pathways.

Another study highlighted the regulatory role of *Nek2* in the MAPK and Wnt/β-catenin pathways^50^. These pathways are also important in osteogenic differentiation. *Nek2*, as a key regulatory molecule, may promote osteoblast differentiation by affecting the activity of these pathways, which further validates the role of *Nek2* in our study. Experimental evidence suggests that troponin-associated protein (*Troap*) plays a key role in regulating cell proliferation in several tumors^51,52^. One study found that *Troap* accelerated glioma progression through the Wnt/β-catenin pathway^51^. Finally, Kinesin family member 14 (*Kif14*) was a mitotic kinesin whose abnormal function was associated with developmental defects in brain and kidney as well as several cancers^53^. Functional enrichment analysis showed that *Kif14* was mainly enriched in 'macroautophagy' and 'pyrimidine metabolism'. In addition, *Kif14* was also active in signaling pathways such as Wnt signaling^54,55^, Hedgehog signaling^56,57^, and PI3K/Akt signaling^58^. To date, there have been no studies on the direct involvement of the above biomarkers in the osteogenic differentiation of MSCs. These studies also suggest their possible cross-cutting roles in the regulation of osteogenic differentiation under the influence of SPA. Thus, the specific mechanisms of biomarkers such as *Asf1b, Troap*, and *Kif14* in osteogenic differentiation need to be further investigated, but their importance in other cellular processes suggests that they may regulate osteogenic differentiation through complex networks and signaling cascades. In addition, the cited literature emphasizes that understanding the complex molecular interactions and signaling cascades in osteogenic differentiation is essential for the development of therapeutic approaches targeting bone-related diseases. Our findings contribute to this knowledge base by suggesting how SPA may regulate these processes through specific molecular markers. In conclusion, we suggest that future studies could further explore the specific mechanisms of these molecules in osteogenic differentiation to better understand the complex network of osteogenic differentiation and provide new ideas for the treatment of bone-related diseases.

To date, studies on the regulation of osteogenic differentiation of BMSCs by non-coding RNAs have been reported. However, there were few studies on the regulation of osteogenic differentiation of BMSCs by non-coding RNAs in SPA mimicking inflammatory environment. From the lncRNA-miRNA-mRNA network in this study, it could be found that miR-497-5p and miR-322-5p had an action relationship with both Asf1b and Nek2. And a study showed that miR-497-5p was significantly downregulated in bone tissue of aging and osteoporosis mouse models and upregulated during osteogenic differentiation of MC3T3-E1 cells. Overexpression of miR-497-5p promoted osteoblast differentiation and mineralization^59^. In addition, a study showed that miR-322-5p was significantly downregulated during osteogenic differentiation of rat BMSCs^60^. Second, miR-455-3p was shown to promote osteogenic differentiation, which may be related to fracture healing^61^, while the present study found a regulatory relationship between miR-455-3p and Troap. Finally, miR-207 was significantly downregulated during FK506-induced osteogenic differentiation of rat BMSCs, while the present study showed an association between miR-207 and Nek2^62^. In conclusion, the present study identified some potential molecular networks of action, the potential significance of which should be clarified by further studies.

Bioinformatics has been widely used for differential analysis of osteogenic differentiation at the genomic level, allowing the identification of functional pathways of DEGs associated with osteogenic differentiation in BMSC. In this study, bioinformatics analysis was performed to obtain some key biomarkers hypothesized to be involved in the regulation of osteogenic differentiation of BMSCs in an inflammatory environment. It provides some clues to explore the key factors of SPA affecting osteogenic differentiation. There were some shortcomings in this study that we would like to highlight. First, we used SPA to simulate the inflammatory environment, which is somewhat different from the real inflammatory environment observed in clinical settings. This simulation may not fully capture the complex interactions present in vivo. In addition, two of the biomarkers identified in our study were not successfully validated, which may be related to the quality of the samples or the limitations of our experimental conditions. This discrepancy highlights the need for further investigation using a larger and more diverse sample set to ensure the robustness of our findings. We also recognize that the bioinformatics approach, while powerful, has its own limitations. It provides a broad overview but lacks the granularity that detailed experimental validation can provide. We are committed to clarifying the roles of these biomarkers through future experiments and to analyzing their molecular mechanisms of action in depth. This will require more sophisticated in vitro and in vivo models to closely mimic physiological conditions and verify the relevance of our bioinformatics predictions.

## Conclusion

Overall, we obtained 6 biomarkers (*Cenpf, Kntc1, Nek2, Asf1b, Troap* and *Kif14*) related to SPA interfered BMSC, which laid a theoretical foundation for exploring the key factors of SPA affecting osteogenic differentiation.

## Data Availability

The raw sequence data reported in this paper have been deposited in the Genome Sequence Archive (Genomics, Proteomics & Bioinformatics 2021) in National Genomics Data Center (Nucleic Acids Res 2022), China National Center for Bioinformation / Beijing Institute of Genomics, Chinese Academy of Sciences (GSA: CRA014184) that are publicly accessible at https://ngdc.cncb.ac.cn/gsa.
